# Gamification and service marketing

**DOI:** 10.1186/2193-1801-3-653

**Published:** 2014-11-04

**Authors:** Roger Conaway, Mario Cortés Garay

**Affiliations:** School of Business, Monterrey Institute of Technology, Campus San Luis, San Luis Potosí, 78216 Mexico

**Keywords:** Gamification, Service business, Customer service, Technology in business

## Abstract

Our paper addresses the development of the gamification concept with business applications. We report on our survey of customers and managers seeking to participate in gamification on their websites. We examined both customer and manager perspectives and compare survey results in terms of service marketing and characteristics of consumers who engage with gamification platforms. Our data supported a design theory delineating four key characteristics in gamification platforms that attract consumers toward an enterprise’s website. Those features attract individuals through (1) Progress Paths, (2) Feedback and Reward, (3) Social Connection, and (4) Attractiveness of the site. Results from the managers’ survey reflected key characteristics that must exist for implementation of a gamification platform. The data revealed a particular demographic profile of a gamification individual drawn to a website. These findings may help company managers who wish to adopt a gamification platform in the future.

**Electronic supplementary material:**

The online version of this article (doi:10.1186/2193-1801-3-653) contains supplementary material, which is available to authorized users.

## Background

The Deloitte Review recently reported on a business trend in service business called Gamification and defined it as “taking the essence of games—fun, play, transparency, design and challenge—and applying it to real-world objectives rather than pure entertainment” (Palmer et al. 2012:54). Similar to the increase of Candy Crush social gaming on smart devices, the Deloitte authors stressed that gamification will be present in 25 percent of redesigned business processes by 2015, “will grow to more than a $2.8 billion business by 2016, and will have 70 percent of Global 2000 businesses managing at least one ‘gamified’ application or system by 2014” (p 54). Bloomberg cites technology research statistics from Gartner to emphasize similar points stating, “a new study by research firm Markets and Markets projects gamification to become a $5.5B market by 2018” (Bloomberg 2014). Assuming growth and acceptance of gamification will occur as predicted, this new development deserves investigation by service business experts in their specialized areas. Gamification seems to merge the activities of marketing and “the thinking of a business manager with the creativity and tools of a game designer” (Palmer et al. 2012:55).

Our paper purposes to quantify the gamification concept from the customer perspective and discover what characteristics engage customers on a company’s website. We sought to identify specific characteristics of customers inclined toward gamification. From the enterprise perspective, we explored characteristics that managers perceive must exist in a business seeking to implement a gamification platform. We first reviewed academic marketing literature on gamification and found scant evidence of academic research on the concept (Herbig 1991; McAfee and McMillan 1996; Vargo and Lusch 2008; Lusch et al. 2007; Reeves and Read 2009; Donato and Link 2013; Terlutter and Capella 2013). Reeves and Read were among the first to assert how gaming is changing the way people work. Other publications appear in computing sciences (see Deterding et al. [2011, a], [b]; Deterding 2012; Huotari and Hamari 2012) and articles about gamification in business were found predominately in trade publications.

In this first section we proceed to address principles of gamification, describe the process as a growing disruptive technology trend, and connect service marketing to gamification. *The Wall Street Journal* identified Badgeville.com as the number one provider of gamification platforms today and the company publishes startling statistics about the positive impact of gamification on the bottom line. Deloitte’s publications added theory about basic elements of gamification, which we briefly review, but most companies appear to assess gamification success upon sales increases, return visits to web sites, customer loyalty, user engagement, or other variables. These methods of assessment involve many variables and only led us to ask additional questions: What characteristics attract consumers toward an enterprise’s website with a gamification platform? Do managers perceive certain basic elements should exist in their companies before gamification can be a success? What implications can be drawn about assessment from a customer perspective of gamification through a survey instrument?

Several reasons exist why answers to these questions are important. First, gamification has become a strategic imperative for leadership in numerous businesses and they seek answers to these questions. Second, businesses gain competitive advantage when adapting to customers and employees in this new digital age. Finally, profit margins may be positively affected by gamification through growth in customer loyalty, sales increases, and increased visits to web sites. Practitioners project incorporation of gamification technology at the highest levels of the enterprise. Gabe Zichermann, CEO of Gamification Co., asserted that gamification must occur at the level of strategic imperative for organizations. He believes the concept “has become a buzzword, for sure, but many enterprises have just scratched the surface of its potential. Over the next year, gamification is likely to morph from a tactical concept to a strategic imperative” (Zichermann 2013). In fact, he promotes in his new book how companies must recruit and retain talent from the ‘gamer’ generation and beyond (Zichermann and Linder 2013). Palmer (2013) adds evidence by stating “over the past year or so, gamification has become a central part of almost every enterprise business package”. Indeed, practitioners and academics alike must work together with the new ‘Digital Natives’ (original term by Marc Prensky 2001) who enter the workplace or classroom with mobile devices in hand.

### Marketing perspectives

The marketing literature reveals negligible evidence of academic research on gamification (see Herbig 1991; McAfee and McMillan 1996; Vargo and Lusch 2008; Lusch et al. 2007; Donato and Link 2013; Terlutter and Capella 2013). In this section we review existing published literature in marketing and extend our review to the information interfaces literature. Finally, we conclude with a review of trade publications and newsletters that address gamification. Initial research on gaming appeared to combine game studies with social sciences that had few business applications. Most research related to educational contexts, social networking, and commercial gaming. Terlutter and Capella (2013) reported on gamification in advertising and analyzed research directions of In-game Advertising (IGA), Advergames and Advertising in Social Network Games. Although the term “Gamification” appeared in the title of their article, the term did not reappear elsewhere and the authors did not address gamification concepts as defined in this current paper. Advergames was described as gaming “specifically designed and created to promote a brand, product, service, or idea” (Terlutter and Capella 2013:96). Their analysis did not include marketing concepts or service marketing connections. They concluded that researchers should add a category of research on advertising in digital games and encourage more research on advertising in social network games.

In early marketing literature on gaming theory, Herbig (1991) provided a comprehensive overview of game theory and asserted that gaming delivered a useful and appropriate tool to define and explain marketing problems. McAfee and McMillan (1996) addressed business competition and game theory and concluded that gaming could be valuable to marketing. They suggested several ways to prove value: motivating a sales force with the design of new selling methods, providing a supplement to the traditional survey approach for assessing the value of new products and new product design, and providing insights into subcontracting and purchasing methods, such as overcoming the disadvantages of sole-source negotiation when competition is possible.

Huotari and Hamari (2012) examined service marketing and gamification in terms of enhancing value in customer service. They defined gamification as “a process of enhancing a service with affordances for gameful experiences in order to support user’s overall value creation” (Huotari and Hamari 2012:22). We adopt this approach and agree with Huotari and Hamari on how this definition anchors gamification into existing service marketing concepts such as service packaging, value in use, and service systems. Huotari and Hamari (2012) attribute, for example, the success of mobile services such as Foursquare to gamification. They define it as a form of service packaging where a core service is enhanced by a rules-based service system that provides feedback and interaction mechanisms to the user with an aim to facilitate and support the users’ overall value creation.

The American Marketing Association introduces gamification as “the process of applying the psychological and sociological factors that drive intense game play to consumer measurement” (Donato and Link 2013:40). The authors suggested four broad guidelines for those implementing gamification when reporting on a pilot study measuring consumer behavior and attitudes of those viewing television on smart phones: “(1) game mechanics should drive competition and reward achievement, (2) techniques work differently across populations, (3) gamification appears to work better with a long-term panel survey than a one-time survey, and (4) techniques should motivate consumers to achieve the measurement tasks but not to drive or change the behavior or attitudes that are being measured. We have incorporated these guidelines in our study except for the long-term panel survey. We assume differences across populations and cultures respond more favorably to gaming techniques than older adults (Terlutter and Capella 2013; Donato and Link 2013).

From an Information Interfaces perspective, Deterding et al. (2011a) believe the term “gamification” originated in 2008 in the digital media industry and became widespread in 2010. They define gamification as “the use of game design elements in non-game contexts” (p 10) and demarcate the concept from playfulness, playful interaction, or design for playfulness, instead referring to a complex of terms as gamefulness, gameful interaction, and gameful design. Thus, business applications may prefer to frame gamification in terms of *gameful* interaction rather than *playful* interaction. Goehle (2013) distinguishes gamification from video games in the educational context and Cohen (2011) argues that online social games may soon replace textbooks in schools. These education trends may prepare employers to incorporate gamification processes in their business processes.

Finally, a search of trade publications and newsletters, including advertising, finance, computer technology, entrepreneurship, and banking, reveal hundreds of short articles, most of which were published from 2011 to 2013. Many of these publications report positive results in revenue growth after the adoption of gamification software in the enterprise. Port (2013), for instance, wrote how Conductor, a New York City-based technology company, had demonstrated a track record of 6,700 percent revenue growth between 2009 and 2012. The challenge was to continue the incredible momentum. The company reported “record revenue growth for the first half of 2013 and a 126 percent annualized increase in sales” after incorporating gamification software and implementing it via the Salesforce.com CRM platform (Port 2013). These types of stories proliferate throughout the trade literature.

In summary, we agree with a caution that “we’re really missing out on some of the elements that make real games compelling when we just boil it down to the leaderboard and achievement levels and badges. If you don’t have the fun factor in there, I’m not sure it really qualifies as a game” Technological Horizons in Education Journal Magazine (2013):33). Thus, we included a questionnaire item addressing “having fun” while on a business website. Gaming also may refer to digital games that have learning objectives. Gamification is digital and will involve points or tokens, badges, levels, rewards, or competition, the same characteristics as gaming, and gamification must also involve fun for the consumer. Gamification differs primarily from gaming in that it involves the customer service experience end-to-end while engaging on a company’s website. Consumer loyalty develops via psychology and game design to change consumer behavior in favor of company objectives. However, if gamification platforms lack novelty and creativity or unexpected twists and difficulty choices for the consumer, they may not endure over the years. They may prove to lose consumers’ interest over time. With gamification, consumers benefit by engaging among themselves about products and services and receive rewards.

The implementation of gamification platforms in business appears to be gaining acceptance as investments in the software programs show high return on investment and greater customer engagement and monetary results. Before briefly discussing service marketing with gamification, one other important distinction must be introduced. The applications of gamification platforms seem to be developing in two different directions. First, numerous organizations are using gamification techniques internally to motivate employees in their performance. Gamification engages and encourages employees to earn vacations and rewards and compete with other employees and is used primarily used with sales personnel. Second, gamification techniques are used externally with customers. Our study focuses on the external dimension of new and returning customers. Before we introduce definitions of gamification and define the concept, we address the context of service marketing.

### Service marketing

We assume gamification is closed connected with service marketing. In this section we build on our review of marketing and information interfaces literature to address service marketing. Broadly, service marketing is defined as those “deeds, processes and performances” that a person or entity provides to another person or entity (Zeithaml et al. 2008). While we agree with this definition, we also acknowledge that the history of business service has evolved to consider all economic activities whose result is not a physical product. Service marketing provides added value in forms that are essentially intangible from that of its first purchaser, and we make a clear reference to gamification and its intangible properties. Four major distinctions between tangible goods and services can be identified and apply to our current study. We assume services are considered intangible because (1) they cannot be inventoried, (2) they cannot be patented or easily displayed, (3) they cannot be communicated easily, and (4) pricing of services is complicated. Services may also be described as “heterogeneous”, implying that service delivery and customer satisfaction depend on the actions of the employee and the customer. Customers do not *buy* gamification; it becomes vehicle for the customer to engage with the company.

Thus, the quality of service of gamification depends on uncontrollable factors. There is no accurate prediction that the service delivered corresponds to what was planned and promoted. Another heterogeneous factor assumes the production and consumption of service is simultaneous. Customers participate in and affect the transaction, drawn in through the gamification platform, interacting with each other. Employees also affect the outcome of the service and adaptation of the company. Decentralization must be essential and there is no mass production. Gamification services have a perishable nature as it is difficult to synchronize supply and demand services because they cannot be returned or resold.

We make one other point about measurement of gamification services. Gamification platforms and services have high levels of expertise and credibility properties, which make them difficult to evaluate particularly before purchase. Normally, the consumer faces three stages in the process of acquiring services. First, consumers recognize their need through information they have received about the company or product. They search for information and evaluate alternatives before the purchase or engagement. Next, the consumer experiences the service. Finally, the consumer evaluates the service after it has been experienced. One goal of gamification is to draw the customer through these three phases and provide an enjoyable experience. These steps require the provider of the service to reflect on the consumer because their active involvement when seeking the service must be considered in each stage. The end result will mean having customers who are willing and delighted to commend the service to others and who become loyal to the service.

Thus, customers interact with employees and often people outside the service environment to produce the final service. We consider gamification a vehicle that engages consumers in the service marketing process. This view is consistent with Vargo and Lusch (2008); Lusch et al. (2007) that participatory customers, embracing the service dominant logic of marketing, promote the idea they are always co-producers of value. Effective participation of customers may increase the likelihood that their needs are met and that the benefits they seek to achieve, in services like health care, education, weight loss, and others, depend on their engagement. Gamification will play an essential role in their participation. Thus, unless customers perform their functions in service marketing effectively, the enterprise cannot get the desired service result. With this characterization of service marketing in mind, we transition to a section on principles of gamification. The following section examines definitions, processes, and elements of gamification.

### Principles of gamification

Various definitions of the gamification concept have been offered by practitioners and published academic literature (Herbig 1991; McAfee and McMillan 1996; Vargo and Lusch 2008; Lusch et al. 2007; Reeves and Read 2009; Donato and Link 2013; Terlutter and Capella 2013). The business advisor for JD Supra Buzz offered a glossary of terms and defined the term as “a business strategy which applies game design techniques to non-game experiences to drive user behavior” (Pierce 2014). *The Wall Street Journal* described top disruptive technology trends in 2014 (Burris 2014) and described gamification as “a fast-moving hard trend of using advanced simulations and skill-based learning systems that are self-diagnostic, interactive, game-like and competitive–all focused on giving the user an immersive experience.” Badgeville, a Redwood California-based company established in 2010 and identified as the number one provider of gamification platforms today, defined gamification as “a proven business discipline that takes the techniques that make games engaging, fun, and compelling, and applies them to technology investments” (Badgeville Vimeo Video 2014).

Furthermore, Badgeville creates platforms for Global 2000 businesses and provides gamification software for such enterprises as Philips Electronics, Kendall Jackson Winery, American Express, Citrix, Bell Media, and Ask.com. The company characterizes gamification as a behavior platform for community engagement (Badgeville 2014). Significant statistics emerge about the positive effect of gamification software on interaction with customers. Badgeville states that 54 percent of customers are inactive in loyalty programs and 69 percent do not use a company’s online communities (Badgeville White Paper 2012). Furthermore, “on average, web-based communities never see more than a 30 percent login rate, while content served up on Facebook reaches only 16 percent of users (many of whom never click on it)” (Badgeville Marketing Campaigns 2014). Interestingly, the company demonstrates that implementation of gamification increases a daily return rate by 33 percent, improves retention by 50 percent, and increases customer advocates by four times (Badgeville Social Loyalty 2014). In summary, these definitions and statistics seem to indicate why gamification platforms are predicted to increase significantly in redesigned business processes and gain presence in Global 2000 businesses. Next, we turn to the gamification process itself and examine several elements that are involved.

### Processes of gamification

Gamification differs from educational learning games and online gaming so prominent today because such activities are unrelated to business organizations and entities serving stakeholders. The gamification process differs from sales promotion programs in marketing that are designed to create brand loyalty with customers. Such frequency or continuity programs focus on customer behavior. Instead, gamification involves the total customer experience, including inward experiences and motivations that are integrated throughout the service marketing process. We offer three illustrations of companies that have incorporated gamification: Starbucks, Allied International Credit, and Samsung.

First, Starbucks illustrates gamification in service marketing with a program called My Starbucks Rewards (Rewards.starbucks.mx 2013). Gamification begins when customers receive a personal Starbucks card at no cost, once they have registered their personal data online. Afterwards, they can recharge the card for about US$4.00 which allows them to automatically move to the Welcome Level. At this level, they will receive a free welcome drink on their next visit plus another drink on their birthday. Once customers have used the card five times, they receive five stars and move from the Welcome Level to the Green Level. After the customers have earned 30 Stars in 12 months (25 additional Stars after starting the Green Level), they qualify for the Gold Level.

Starbucks has increased customer engagement, developed stronger customer loyalty, and increased sales as a result of My Starbucks Rewards. Gamification directs customers toward greater motivation and “to move to the next level”. The gaming process creates participation in the process itself and the game mentality begins. Customers may track their “stars” online by logging into their account. The exchange of any free drink or redemption, such as syrup or free milk, does not advance the stars program, but the gamification condition exists in that the service in the store has to be evident to invite costumers to go forward in obtaining stars and to go to the next level.

A second example illustrates gamification in financial services. King (2013) relates how a collections agency, Allied International Credit, a unit of Allied Global Holdings Inc. based in Newmarket, Ontario, motivates its customer service reps by using gamification. The company allows employees to earn points and prizes for routine job tasks through gamification. King explains how approximately 100 agents at Allied International “accrue points and badges and they can see where they stand relative to other call center workers. Sometimes, the feedback is just a fun badge when they call a new city for the first time. There is a maximum of 5,000 points that can be earned each day and a number of different prizes” (King 2013). Moreover, employees can win $5 coffee cards, time off on the Friday before a holiday weekend, dinner with the CEO, or golf with an executive vice president. Gamification helps motivate new employees who on average last only six months at the collection agency and help make collections more interesting for the more experienced employees.

One final example illustrates the gamification process at Samsung.com. Badgeville provided the technology platform for Samsung, an engagement program called Samsung Nation, a program which rewards behaviors that require customer time and attention. Samsung, for example, created user-generated content by providing the following gamification strategy (adapted from Badgeville White Paper 2012):

500 pts Register Samsung products
300 pts Provide answers in Q&A
300 pts Submit comments and reviews
200 pts Watch videos
200 pts Facebook “likes”
100 pts Share on Twitter
100 pts Provide questions in Q&A

Not all Samsung customer behaviors are treated equally. According to the company’s website, providing an *answer* in the Q&A is worth more than merely *asking* the question. The idea of points and rewards is to increase customer engagement and provide rewards for their behaviors.

### Elements of gamification

**Figure 1:**
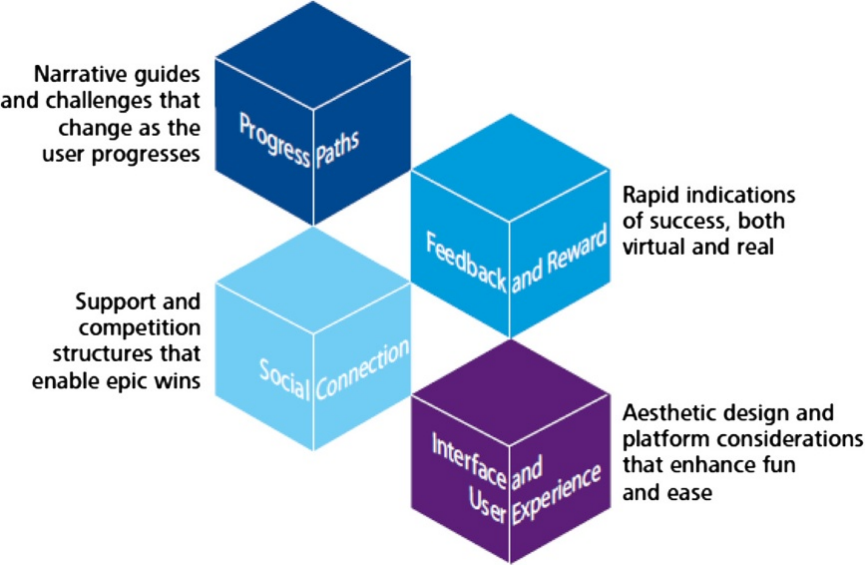
**Visual display of gamification elements.**

Palmer et al. (2012) have distilled game-mechanics principles, behavioral economic theories, and current user experience design thinking into four gamification elements. They describe the elements and tools of the gamification process in the Figure 1 (adapted from Palmer et al. 2012:56).

1. The first element, Progress Paths, is identified as “the use of challenges and evolving narratives to increase task completion” (Palmer et al. 2012:56). Typically, gamification begins with an easy task and then progresses to more complex challenges over time. A novice is rewarded and more advanced users stay engaged in the process. Thus, the progress path becomes more complex as the user progresses.
2. The second element, Feedback and Reward, are defined as “the use of rapid indications of success through virtual and monetary rewards” (Palmer et al. 2012:56). Gamification typically rewards participants instantly, using traditional monetary rewards, but other use delayed gratification depending on the path the customer takes. Some customers desire to have a level of power, leadership or responsibility as they progress.
3. The third element, Social Connection, leverages a customer’s social networks to create competition and provide support. Many gamification programs provide instant access to friends and social connections, serving as a key element of the attraction to gamification.
4. The fourth element, Interface and User Experience, must be attractive to users in terms of video game graphics and web page design. For small and medium enterprises, such sophisticated design requirements can pose a challenge for an organization with limited staff.

### Applications in service marketing

Many of these elements can be identified in gamification platforms. Although loyalty programs also are consumer oriented, loyalty differs from gamification because of its narrow focus on behavioral rewards. Typically, a loyalty program rewards customers who make frequent purchases with privileged access to various company services. These privileges may include discounts, special services, coupons, or free products, but they benefit the company by giving intense marketing information about the customer. In contrast, gamification involves the entire process of service marketing and emphasizes inward motivation through different levels. The Wall Street Journal pointed to how “Gamification isn’t just about scoring points—it allows for new ways of imagining, designing, and implementing solutions. As business becomes increasingly social, more opportunities are arising to augment performance and promote strategic objectives by embedding gaming mechanics into traditional processes” (Palmer and Hugo 2013).

We assume that elements 1 and 2 mean gamification behavior differs across demographics. Depending on the age of the participant, we must take into account the engagement techniques of frequent consumers. They obtain more from the firm and recognize in their environment how companies have to improve their marketing service for them to draw them in.

### Research questions

Gamification appears to be an accelerating technology trend in business service that incorporates gaming appeals to the new “digital native” generation. The billion dollar industry is growing at a rapid rate and academic marketing research has not kept pace. We proposed these research questions for our investigation of the gamification concept within the Latin American and the U.S. market:

- RQ1: What characteristics attract consumers toward an enterprise’s website that seeks to engage them online?
- RQ2: What implications can be drawn about assessment of gamification from a customer perspective?
- RQ3: What important characteristics do Latin American managers perceive to exist to incorporate gamification techniques in their enterprises?

## Method and results

### Participants

Two populations of data were selected for our customer survey and we obtained a sample of respondents (N = 189). The first population were registered users of Amazon’s Mechanical Turk (MTurk) web service that makes available approximately 500,000 participants from 190 countries. According to Buhrmester et al. (2011), MTurk is a new source of high-quality, inexpensive data. They found “MTurk participants are slightly more demographically diverse than are standard Internet samples and are significantly more diverse than typical American college samples” (p 4). A convenience sample of respondents, who voluntarily and anonymously completed the questionnaire (N = 130), was obtained in June 2014. Individuals who chose to participate in the study received after survey completion a small monetary award, which was deposited into their MTurk account. This survey was posted in English. A second convenience sample of customer respondents (N = 59) was taken from our university campus student population during May 2014. The customer survey was posted online in Spanish for university student and professor access through a link on our School of Business Facebook page. An announcement was posted for professors and students asking them voluntarily and anonymously to participate in the study. The questions and scaling for both the MTurk survey and the Facebook survey were identical, differing only in language translation.

### Survey development

Typically, assessment of gamification activities is based upon growth in sales revenue, retention of customers, or increased return of customers on company website. These particular variables are no doubt important to businesses, but each variable can depend on multiple factors. Sales revenue increases, for instance, may be based on product pricing or customer preferences rather than gamification. To date no published instruments or surveys appear in the indexed scientific literature that addresses or quantifies gamification. As a result, our study involved the development of a 20-item questionnaire measure that targeted customer engagement on company websites. Reliability tests on the combined samples of the customer survey revealed Cronbach’s α = .913.

Items first were pilot tested with young entrepreneurs associated with our university and who used a gamification platform on their websites. The 20-item questionnaire (see Additional file 1) asked respondents to consider their LAST experience with a business where they received a special promotion, a discount, or used a membership card to obtain a service at the business, and to write the name. The next three items assessed how often the respondent visited the company website, length of time as a customer, and how many times a month the respondent visited the web site. Following items addressed gamification-related topics, such as the experience of having “fun” while visiting the website, the presence of a special “feeling” toward the company while spending money, and obtaining additional benefits with purchases.

A second separate survey (Additional file 2) was developed to assess management perspectives related to gamification and the degree to which these managers believed they met consumer needs and delivered benefits. The 22-item instrument contained 19 items related to perspectives about loyalty of customers, resources available for implementation of new technologies, website attractiveness, and customer engagement. The analysis included questions 1–16, which used Likert-type scaling of 1 = strongly agree to 5 = strongly disagree. The survey items were developed from company publications about gamification available through website searches. Three demographic questions appeared at the end and addressed gender, age, and level of education. Managerial data were gathered (N = 15) through a paper survey distributed at a managers’ meeting of a local restaurant group in April 2014. Reliability tests revealed Cronbach’s α = .521.

## Results and discussion

### Customer survey independent samples T-Test

An independent samples T-test (SPSS 2014) was conducted on the MTurk and online Spanish samples on the three demographic variables of age, gender and level of education. Age and gender variables revealed significant differences in the two samples, while level-of-education was not significant (Table 1).

**Table 1:** **Independent samples T-Test**

Demographic items	Results		
Levene’s test for equality of variances	F	Sig.	***t***
Q18 age	36.118	< .000	10.329
Q19 gender	13.606	< .000	17.507
Q20 level of education	1.885	< .172	9.685

Further examination of these variables is displayed in Table 2. Different levels of demographic variables shows 100% of the participants in the MTurk survey were 24 years old or less, while approximately half of the Spanish survey participants were around 24 years old. Comparisons of gender in the MTurk survey approximated the percentages reported by Buhrmester et al. (2011). The gender percentages in the Spanish survey reflected two-thirds female and one-third male. Although the independent samples *t* resulted in sig < .172, examination of Table 2 shows MTurk participants with much less education than Spanish survey university participants. Thus, a profile of the average gamification participant seems to be female, around 24 years of age, and having at least a pre-university education. Note the frequencies in Table 2 adding to less than the sample amount were missing cases.

**Table 2:** **Demographic comparisons**

Gender	MTurk		Spanish	
	Freq.	%	Freq.	%
Female	69	58.5	16	28.1
Male	49	41.5	41	71.9
Totals	118	100%	59	100%
**Age**	**MTurk**		**Spanish**	
	Freq.	%	Freq.	%
Under 20 years	48	41.4	3	5.2
20 – 24	68	58.6	24	41.4
25 – 35			19	32.8
36 – 49			7	12.1
50 and over			5	8.6
Totals	116	100%	58	100%
**Level of education**	**MTurk**		**Spanish**	
	Freq.	%	Freq.	%
Less than high school	19	16.4	1	1.7
High school graduate	50	43.1	2	3.4
Technical school graduate	22	19.0	2	3.4
Some university or college	12	10.3	23	39
University or college graduate	6	5.6	15	25.4
Advanced degree	6	5.6	16	27.1
**Totals**	115	100%	59	100%

### Factor analysis

To identify possible underlying dimensions in the customer survey, factor analysis was conducted on the combined customer data sets (N = 189). Principal components extraction with Varimax rotation resulted in two components or factors emerging with an eigenvalue >1 and accounting for 61 percent of the variance. The analysis included questions 5–17, which used Likert-type scaling of 1 = strongly agree to 5 = strongly disagree. Thus, a total of 13 questions were analyzed, surpassing the 5:1 ratio of cases to variables requirement in a principal components analysis.

We named the first dimension, *Relationship and Reward,* based on four questions loading above 0.75 and explaining 49.1% of the variance. Dimension two, which explained 11.8% of the variance, was named *Competition and* Motivation and contained four questions that loaded above 0.75. Table 3 reflects the eight key questions and their significant loadings.

**Table 3:** **Dimensions of customer gamification**

Survey items	Cronbach’s α = .913	
***Relationship and reward*** **(first dimension)**	Factor loadings	Means
Q5 My relationship with that business is excellent	0.860	4.0
Q6 I have a membership or frequent customer card or related membership.	0.885	4.0
Q7 I receive special treatment with my membership or frequent client card.	0.804	3.8
Q8 The more money I spend in the business, the more benefits I obtain.	0.778	3.9
***Competition and fun*** **(second dimension)**		
Q12 The company’s website attracts me because it gives me an opportunity to earn benefits.	0.721	3.7
Q13 The company’s website allows me to move to higher levels of participation to receive more benefits.	0.820	3.7
Q14 I like being the best among my friends with gaming activities.	0.738	3.6
Q15 I have fun when I spend time on the website.	0.769	3.7

Our factor analysis revealed that business relationship, rewards, competition, and fun were fundamental driving dimensions in gamification. Interestingly, respondents did not indicate receiving different treatment (Q7), obtaining more benefits when spending money (Q8), receiving different treatment in that business with a card (Q9), feeling something special (Q10), and spending more money to receive excellent treatment (Q11) were important factors. We initially assumed that “special feelings” and “moving to higher levels” would emerge as key dimensions of gamification. Moreover, the final two questions, “I enjoy sharing my activities with my friends on social media” (Q16) and “The benefits that are offered are easy to understand” (Q17), did not emerge as significant items in the analysis. Although Q16 “sharing of activities” loaded at .500, we initially assumed respondents would indicate sharing on social media of stronger importance.

### Crosstabs

Results from frequency analysis of visits to business (Q2); length of time as a customer (Q3); and number of days, weeks, or months using the company website, brought out key patterns of gamification. Over half of the respondents visited their business once a week or more (34%) or once every two or three weeks (20.9%). Another one-fourth indicated once a month. When asked how long the respondent had been a customer of the business, almost two-thirds (63.1%) indicated one year or more. Finally, when asked how many times a month they accessed the company website, 11.2% indicated once a day, 18.7% two or three times a week, and 15% accessed the site once a week. Thus, 45% of the respondents could be considered frequent visitors to their business web site. We assume frequency of visits and engagement would be instrumental in the gamification process.

Cross tabulations were conducted with each question item and each demographic variable. Results showed the Gender was significant with Relationship with the Business (Q5) at Pearson’s Chi-Square value 130.391, df 20, p < .000; Level of Education was significant with Relationship with the Business, value 67.020, df 24, p < .000; and Age, value 63.190, df 16, p < .000. These results again emphasize the importance of business relationship with gamification. Other question items having significant Pearson’s Chi-Square values (confidence level p < .05) on all three demographic variables were: having membership and frequent customer cards (Q6), more money equals more benefits (Q8), sharing my activities with my friends on social media (Q16), and benefits are easy to understand (Q17).

### Manager survey results

Fifteen managers who were part of a large restaurant group in our city were surveyed for their perspectives on gamification in May 2014. They formed a leadership team of their businesses and had expressed interest in gamification platforms. The survey was administered by paper during a monthly meeting and the group was given assurances of confidentiality of the data. Table 4 reflects means of each variable on the survey. Results revealed Q4, Q6, Q11 and Q14 had the highest means, implying customer satisfaction, customer loyalty, free time at work, and sufficient funding were most important factors if the company implemented a gamification platform.

**Table 4:** **Means of manager survey**

Questions 2 – 4		Chi-Square Tests		
		Means	df	Means
Q1	My business has grown steadily during the last 5 years.	1.80	Q11 My workers believe they have “free time” while at work.	3.50
Q2	I believe I can increase sells during the next 6 months.	1.40	Q12 My workers are willing to learn or be recruited to learn new services marketing techniques.	2.00
Q3	My business tends to have loyal clients who visit more than once.	1.27	Q13 My intent is to implement new technologies to sell more products or services.	1.64
Q4	My consumers feel satisfied with my products.	2.53	Q14 I have enough funds to implement new loyalty programs.	2.57
Q5	My clients obtain positive experiences when they are in contact with my products.	2.20	Q15 My consumers use our company’s website often.	2.29
Q6	My consumers tend to feel they are part of my company.	2.60	Q16 Our company’s website attracts consumers because it gives them an opportunity to earn benefits.	2.14
Q7	I understand the specific needs of my clients.	1.93	Q17 Our company’s website allows consumers to move to have different levels of participation to receive more benefits.	2.36
Q8	If I give my consumers more value, they are willing to pay more for it.	1.73	Q18 Our company website is attractive to consumers.	2.07
Q9	My products are the most preferred in my competitive market.	1.60	Q19 Our website engages consumers.	1.71
Q10	I have loyal workers who work with my company for long periods of time.	2.07		

Table 5 reflects the demographics of the Latin American manager’s sample. On average, the respondents appear to be middle aged, university educated, and predominately female. Over 86 percent indicated ages between 25 and 49 years old.

**Table 5:** **Demographics of manager sample**

Gender	Freq.	%
Female	9	60
Male	6	40
Totals	15	100%
Age	**Freq.**	**%**
Under 20 years	0	0
20 – 24	2	13.3
25 – 35	8	53.3
36 – 49	5	33.3
50 and over	0	0
Education	**Freq.**	**%**
Less than high school	3	20.0
High school graduate	1	6.7
Technical school graduate	1	6.7
Some university or college	4	26.7
University or college graduate	5	33.3
Advanced degree	1	6.7

## Conclusions

Gamification appears to be an exploding business trend that will be a multi-billion dollar market 2018. It seems to be “a fast-moving hard trend” and platforms have been incorporated into business websites at a rapid pace, according to *The Wall Street Journal*. Various definitions of gamification have appeared in the trade and academic literature, but the simplest one seems to be “a business strategy which applies game design techniques to non-game experiences to drive user behavior” (Pierce, 2014). As discussed in the review of literature, Badgeville provides a definition focused on technologies as “a proven business discipline that takes the techniques that make games engaging, fun, and compelling, and applies them to technology investments”. Each of these perspectives led us to include items in our questionnaire about fun, feeling, competitiveness, and rewards.

Our first research question addressed the topic of what characteristics attract consumers toward an enterprise’s website and engage them online. Our findings revealed significant characteristics that attract and engage of customers. Data taken from two different customer populations revealed key items of the presence of strong business relationship, presence of rewards, increased competition, and having fun, characteristics which businesses may utilize when considered gamification software. Furthermore, Palmer et al. (2012) elements appeared to integrate well with these characteristics.

Our analysis produced an underlying dimension labeled Competition and Fun that combine the first and fourth elements of visual design, Progress Paths, which are challenges to increase task completion, and Attractiveness of the web site’s interface and user experience, which must provide “aesthetic design and cross-platform integration considerations to enhance fun” (Palmer et al. 2012:57). Additionally, a second underlying dimension was revealed labeled Relationship and Reward. This dimension combined the second element, Feedback and Rewards, which is utilizing rapid indications of success, a necessity in the Prensky’s (2001) digital native generation, and the third element, Social Connections, although leveraging social networks with mobile phones or tablet devices to create competition and share the results did not emerge as significant as the other characteristics. These findings may enable company managers to effectively adopt a gamification platform for their particular enterprises.

Next, the second research question addressed what implications can be drawn about assessment of gamification from a customer perspective. Primarily, consumers will engage with websites that begin with an easy task and then progresses to more complex challenges. They will want rapid indications of success through virtual and monetary rewards. Possibly, customers will forego these instant awards if they obtain a level of power, leadership, or responsibility as they progress, such as taking part in decision making or influencing a product’s design. Many, but not all consumers, will want to use their social networks to create competition and provide support. Finally, and possibly the strongest implication that can be drawn, is that the website must be attractive to users in terms of video game graphics and web page design. Thus, assessment of gamification could focus on the degree to which these characteristics are present in their customers and could measure the impact of their contributions and participation on the website.

Interestingly, the data seemed to reveal a particular demographic profile of a gamification consumer who is drawn to a website. We concluded from our demographic data that an average profile of an individual drawn to a website would have a long term relationship with the business, is female around 24 years old, have at least a pre-university education, and would visit the business web site frequently. As difficult and controversial as profiling may be, doing so may serve to frame discussions of gamification markets and offer ideas for future research.

Finally, our third research question asked what characteristics Latin American managers perceive should exist in their enterprises to incorporate gamification techniques. Based on the sample’s demographics, we concluded that the managers were mid-career professionals who were young enough to be familiar with new technologies. Their education combined with experience led us to believe they understood business strategy and processes and could comment knowledgeably about adopting new technology platforms. Four questions receiving the highest means from the managers’ survey indicated they believed consumers were satisfied with their products (Q4), felt customers were a part of their companies (Q6), thought their workers had “free time” while at work (Q11), and believed their companies had enough funds to implement new loyalty programs (Q14). For a new gamification platform to be installed, the enterprise would need to have strong customer relationships and overall customer satisfaction with their products. Internal to the enterprise, managers would need to give employees extra time and funding to implement the platform. The available time for workers emerged as the strongest variable (mean 3.5) to be in place for implementation of a gamification platform.

Significant limitations of our study appeared in at least two areas. First, the questionnaire for customers must be refined and tested further for validity and reliability. Few scientific studies have provided sufficient theory or direction to develop a measuring instrument for gamification. The items for the survey used in this study were derived from characteristics described in trade publications and company periodicals, apparently our only source of business information. Future researchers may advance research by refining both customer and manager measuring instruments for specific industries. Another limitation involved the internet samples. Each sample was broad, and future samples could focus on specific industries or sectors of retail service businesses. Although our samples were taken in two different cultures and from two different populations, the single simple t-test showed significant differences from the population mean.

## Electronic supplementary material

Additional file 1: **Customer Survey.** (DOC 36 KB)

Additional file 2: **Manager Survey.** (DOC 36 KB)
