# Supplementary material for: Gamification and service marketing
Source: Springerplus. 2014 Nov 4;3:653. doi: 10.1186/2193-1801-3-653 (PMC4227984; doi:10.1186/2193-1801-3-653)
Supplement: Supplementary file 1 — Additional file 1: Customer Survey. (DOC 36 KB) [file 40064_2014_1347_MOESM1_ESM.doc]

Appendix A

CUSTOMER SURVEY

Please do not write your name on this survey. Your answers are completely confidential, so please answer each question as honestly as possible. This is not a test and your opinion is the only right answer. No other person will see your individual answers. Survey results will be reported only in collective or group form so that answers cannot be attributed to any individual.

**INTRODUCTION**: Consider your last experience with a business where you received a special promotion, received a discount, or used a membership card to obtain a service at the business. Answer the following questions with that same business in mind.

1. Please write the name of the business. ___________________________________

2. How often do you visit that business?

| Once a week or more | Once every two or three weeks | Approximately once a month | Two or three times a year | Only once a year or less |
| --- | --- | --- | --- | --- |
| 1 | 2 | 3 | 4 | 5 |

3. How long have you been a client or customer of that business?

| One month or less | Less than 3 months, more than 1 month | Less than 6 months, 3 months or more | Less than 1 year, 6 months or more | One year or more |
| --- | --- | --- | --- | --- |
| 1 | 2 | 3 | 4 | 5 |

4. How many times a month do you use the company website?

| At least once a day | Two or three times a week | Once a week or less | Once every two or three weeks | One time a month or less |
| --- | --- | --- | --- | --- |
| 1 | 2 | 3 | 4 | 5 |

**Please indicate the degree to which each of the following statements applies to you by writing the number showing whether you:**

| **1 = Strongly Agree** | **2 = Agree** | **3 = Neutral** | **4 = Disagree** | **5 = Strongly Disagree** |
| --- | --- | --- | --- | --- |

**Write the correct number in the blank space.**

_____ 5. My relationship with that business is excellent.

_____ 6. I have a membership or frequent customer card or related membership.

_____ 7. I receive special treatment with my membership or frequent client card.

_____ 8. The more money I spend in the business, the more benefits I obtain.

_____ 9. I perceive different treatment in that business with my card than if I did not have the card.

_____ 10. I feel something special when I spend more money and obtain more benefits in the business.

_____ 11. I am inclined to spend more money in the business to receive excellent treatment.

_____ 12. I use the company’s website often.

_____ 13. The company’s website attracts me because it gives me an opportunity to earn benefits.

_____ 14. The company’s website allows me to move to higher levels of participation to receive more benefits.

_____ 15. I have fun when I spend time on the website.

_____ 16. The benefits that are offered are easy to understand.

| **17.** **Ethnic Group**   - Latino o Latina - African American - Caucasian - Asian American/Pacific Islander - Spanish (from Spain) - Other   **18. Gender**   - Male - Female | **19. Age**   - under 20 years - 20 – 24 - 25 – 35 - 36 – 49 - 50 and over   **20. Level of Education**   - Less than High School - High School Graduate - Technical School Graduate - Some University or College - University or College Graduate - Advanced degree |
| --- | --- |
